# How traumatic is breast cancer? Post-traumatic stress symptoms (PTSS) and risk factors for severe PTSS at 3 and 15 months after surgery in a nationwide cohort of Danish women treated for primary breast cancer

**DOI:** 10.1038/sj.bjc.6606073

**Published:** 2011-01-11

**Authors:** M O'Connor, S Christensen, A B Jensen, S Møller, R Zachariae

**Affiliations:** 1 Psychooncology Research Unit, Institute of Psychology, Aarhus University, Jens Christian Skousvej 4, Aarhus C 8000, Denmark; 2 Department of Oncology, Aarhus University Hospital, Norrebrogade, Aarhus C 8000, Denmark; 3 Danish Breast Cancer Cooperative Group, Copenhagen University Hospital, Afsnit 2501, Blegdamsvej 9, Copenhagen Ø 2100, Denmark

**Keywords:** post-traumatic stress symptoms, distress, PTSD, breast cancer, cohort studies, prospective studies

## Abstract

**Background:**

The literature shows considerable between-study variation in the prevalence of post-traumatic stress symptoms (PTSS) among women with breast cancer. Our aim was, therefore, to explore the prevalence of and risk factors for cancer-related PTSS in a nationwide inception cohort of women treated for primary breast cancer.

**Methods:**

In all, 68% of all Danish women receiving surgery for primary breast cancer between October 2001 and March 2004 completed a questionnaire at 3 months post surgery (*n*=3343), which included the impact of event scale (IES). In all, 94% of the disease-free women also completed a follow-up questionnaire at 15 months post surgery. Data on pre-cancer demographic, socioeconomic, and psychiatric status were obtained from national registries. The Danish Breast Cancer Cooperative Group and surgical departments provided information on disease variables, treatment, and comorbidity.

**Results:**

At 3 months post surgery, 20.1% had IES total scores suggesting severe PTSS (⩾35), compared with 14.3% at 15 months. In all, 48% with severe PTSS at 3 months also had scores above the cutoff at 15 months. Main predictors of severe PTSS at 15 months were low social status, previous physical and mental illness, axillary lymph node involvement (>3), and reduced physical functioning (PF) at 3 months.

**Conclusion:**

The results confirm that receiving a breast cancer diagnosis can be a significant traumatic experience, and that many women experience persistent cancer-related PTSS. Low social status, poor health status, low levels of PF, and disease severity were found to be risk factors for severe PTSS.

A breast cancer diagnosis is a potential life-threatening event associated with significant distress (Kunkel and Chen, 2003; Somerset *et al*, 2004). Even after successful treatment, cancer diagnosis and treatment may continue to be a source of distress (Mehnert and Koch, 2007). Post-traumatic stress can occur after an individual is exposed to an event perceived as life threatening, and associated with intense fear, helplessness, or horror (American Psychiatric Association, 2000). In 1994, the trauma criteria of post-traumatic stress disorder (PTSD) in DSM-IV were expanded to include life-threatening illness, such as cancer (American Psychiatric Association, 2000). According to DSM-IV, the disorder is defined by a set of symptoms (re-experiencing, avoidance, and hyper-arousal) lasting at least 1 month.

There is considerable variation in the proportion of individuals exposed to traumatic events who develop post-traumatic stress symptoms (PTSS) of sufficient severity to warrant a diagnosis of PTSD (Passik and Grummon, 1998; Smith *et al*, 1999; Gurevich *et al*, 2002). This is also the case for studies focusing on the prevalence of PTSS in breast cancer (Hegel *et al*, 2006; Mehnert *et al*, 2009), with reported prevalence of suspected or diagnosed PTSD ranging from 32% in a sample of 31 women with stage I–III breast cancer on average at 16 months after treatment (Naidich and Motta, 2000) to 0.0% in a sample of 74 breast cancer survivors at 3 to 6 years after diagnosis (Matsuoka *et al*, 2002). As suggested in reviews (Gurevich *et al*, 2002; Kangas *et al*, 2002; Matsuoka *et al*, 2002), this variability may stem from several between- and within-study differences in research designs. First, the available studies mostly involve relatively small samples of convenience, ranging from 31 (Naidich and Motta, 2000) to 352 women (Han *et al*, 2002), with one cross-sectional study including 1083 women (Mehnert *et al*, 2009) as the exception. Second, most studies have used cross-sectional designs (e.g., Hegel *et al*, 2006; Mehnert *et al*, 2009). Third, the timing of assessment varies considerably, from a few weeks to 23 years after treatment (Cordova *et al*, 1995; Baider and Kaplan, 1997; Butler *et al*, 1999; Andrykowski *et al*, 2000; Baider *et al*, 2000; Vickberg *et al*, 2000; Kornblith *et al*, 2003). Fourth, the assessment instruments and diagnostic criteria vary, although more than half of the studies have used the 15-item impact of event scale (IES; Horowitz *et al*, 1979). Finally, although younger age, lower income, less education, physical and psychiatric co-morbidity, and physical functioning (PF) have been proposed as general risk factors of severe post-traumatic stress (Kessler *et al*, 1995; Ullman and Siegel, 1996; Luecken *et al*, 2004; Palmer *et al*, 2004; Palyo and Beck, 2005; Hegel *et al*, 2006; Schlich-Bakker *et al*, 2009), only few studies have conducted multivariate data analysis while adjusting for potential confounders (e.g., age; Cordova *et al*, 1995; Green *et al*, 1998; Kornblith *et al*, 2003).

Although the available results suggest that women treated for breast cancer may be at increased risk of cancer-related post-traumatic stress, the considerable variability in the prevalence of PTSSs the literature calls for large, population-based studies, addressing the methodological limitations of the available studies. We, therefore, assessed PTSS and the role of several potential risk factors in a large nationwide cohort of Danish women treated for primary breast cancer at 3 months after surgery, and again at 12 months later. Our study is, to the best of our knowledge, the first nationwide questionnaire-based prospective study of post-traumatic stress in cancer patients.

## Patients and methods

### Study design

The study was designed as a nationwide cohort study of 4917 Danish women with primary breast cancer who underwent surgery between October 2001 and March 2004, and were treated in accordance with the standardised guidelines provided by the Danish Breast Cancer Cooperative Group (DBCG). Details concerning the cohort have previously been published (Christensen *et al*, 2009). The eligible women were informed about the study at the surgical departments, and the Charlson comorbidity index (CCI; Charlson *et al*, 1987) was completed for each patient. Information on eligibility, comorbidity, histopathology, and treatment were obtained directly from the surgical departments, and/or from the DBCG and the CPR registry. All Danish residents have a unique 10-digit personal identification number (CPR number), which is used across all public registration systems, making linkages between various registry-based databases possible. Demographic data, psychiatric history and socioeconomic variables were collected from six nationwide Danish registries through a linkage serviced by Statistics Denmark (Statistics Denmark, 2010). The study was approved by The Regional Science Ethics Committees and The Danish Data Protection Agency.

### Inclusion criteria

Eligible patients at 3 months post surgery were 18–70 years old resident Danish women with histologically confirmed breast cancer T1-3, N0-3, and M0 according to the tumour–node–metastasis staging system (Singletary *et al*, 2002), and no history of other cancers, except non-melanoma skin cancer or carcinoma *in situ* of the cervix uteri. Because of the considerable variation in the treatment procedures for patients not receiving standard treatment, only women eligible for the DBCG treatment protocols during the study period were included, thus excluding women younger than 18 or older than 70 years. Additional requirements were: ability to read Danish and being capable of completing a questionnaire. Eligible patients at 15 months post surgery were disease free as verified by the DBCG.

### The questionnaire cohort

At approximately 3 months post surgery, 3343 (68.0%) women provided an additional information regarding health behaviours, health status and a number of psychosocial variables through a mail-out questionnaire. Responders were slightly younger than non-responders (median 55.7 years *vs* 58.0 years; range: 26–70 years), but age-adjusted analyses showed the sample to be nationally representative with respect to disease- and treatment-related variables (Christensen *et al*, 2009). At 15 months post surgery, 166 women had either died or suffered a recurrence, and were excluded. On the basis of subsequently reported information, two women did not meet the baseline inclusion criteria and were excluded together with 47 women with unknown disease status at 15 months post surgery. A total of 3128 women were eligible 15 months post surgery. Of these, 2931 (93.7%) returned a valid questionnaire.

### Measures

Post-traumatic stress symptom was assessed with the IES (Horowitz *et al*, 1979). Although not a measure of PTSD *per se*, which is diagnosed through clinical interviews, the high correlations found between the IES and PTSD identified through clinical interviews have supported its usefulness as a measure of post-traumatic symptom severity (Neal *et al*, 1994; Sundin and Horowitz, 2002, 2003; Brewin, 2005), and the IES has been used in the majority of previous studies of women with breast cancer. In addition to a total IES score, the IES yields scores on the two subscales measuring seven intrusive symptoms and eight avoidance symptoms relative to a specific stressor, in this case breast cancer. Possible scores range from 0 to 75 (IES total), 0 to 35 (intrusive symptoms), and 0 to 40 (avoidance symptoms). The cutoff levels for the IES have not been determined in cancer patients, but a cutoff of ⩾35 has demonstrated high sensitivity (0.89) and specificity (0.94) for PTSD when validated against the criteria of DSM-IV, as determined by a clinical diagnostic interview (Wohlfarth *et al*, 2003). It should be noted that the IES was developed before the latest revision of PTSD in 1994, and thus does not include the hyper arousal dimension added in the present diagnostic criteria of PTSD in the DSM-IV (American Psychiatric Association, 2000). We therefore chose to apply the scale conservatively as a broad indicator of PTSS, and not a direct measure of PTSD. Consequently, in the following, the IES total scores will be labelled PTSS, whereas the cutoff of ⩾35 on the total IES score will be labelled as severe PTSS (yes: ‘1’, no: ‘0’). The internal consistencies (Cronbach's *α*) of the IES in this study were satisfactory (total: 0.89; avoidance: 0.88; intrusion: 0.81).

Covariates included the demographic and socioeconomic variables: age at time of surgery, marital status, number of children, educational level, social status, personal income, net-wealth, urbanicity, ethnicity, and psychiatric history, using relevant national registries (Christensen *et al*, 2009). These variables refer to pre-cancer conditions either in the year before the surgery minus 1 month or, when appropriate, the date of surgery minus 1 month. Additional covariates included the weighted index score of the CCI (Charlson *et al*, 1987; Extermann, 2000a, 2000b), the PF subscale of the MOS short form (SF-36; Ware and Sherbourne, 1992), the body mass index (BMI), smoking habits, and alcohol use.

### Missing values

On subscales with ⩽50% missing values and Cronbach's *α* >0.7, missing values were substituted with the mean of the remaining filled items on the subscale (Schafer and Graham, 2002). Subscale scores with >50% missing values were coded as missing and no total score was calculated. This procedure is identical to the procedure described in the SF-36 manual (Bjørner *et al*, 1997) and is regarded as preferable to procedures such as list-wise deleting or scale mean substitution of scale scores (Schafer and Graham, 2002).

### Statistical analysis

As the IES data were highly skewed, unadjusted comparisons between independent variables and continuous IES scores (PTSS) were conducted using non-parametric tests (Mann–Whitney or Kruskal–Wallis) and the unadjusted comparisons between independent variables and severe PTSS with *χ*^2^ tests. The adjusted analyses were conducted with logistic regression analyses, with severe PTSS (yes: ‘1’, no: ‘0’) as the dependant variable. Results are presented as adjusted odds ratios (ORs).

The data were analysed according to the three phases of the woman's cancer history. Demographic factors and health status were analysed at the first step, as these data refer to pre-cancer conditions and, therefore, are unbiased by the cancer experience. Information about the disease and treatment had been known to the women for more than 2 months when the questionnaire was completed, and the role of clinical variables was therefore analysed at the second step. Post-treatment psychosocial and health-related variables measured at 3 month post surgery could potentially be moderated by the women's knowledge of the severity of their specific cancer diagnosis and treatment, and were therefore analysed at the third step.

Associations between baseline covariates, PTSS, and severe PTSS were analysed both at 3 months and 15 months post surgery. At the first step, univariate associations between potential predictors and PTSS and severe PTSS were assessed. Then, as both PTSS and many predictors varied with age, associations were adjusted for age. Finally, we explored predictors for severe PTSS at 15 months post surgery, adjusting for 3-month scores of PTSS and severe PTSS. At steps 2 and 3, the univariate comparisons, both adjusted and unadjusted, were conducted as in step 1. In addition, we also conservatively adjusted for variables associated with severe PTSS (*P*<0.25) at the previous steps. Age was treated as a continuous variable in all multivariate analyses. Analyses were conducted with SPSS 16.0.2 for Windows Server 2003 R2 (SPSS Inc., Chicago, IL, USA).

## Results

### Prevalence

The IES scores (PTSS) were imputed for 117 women at 3 months post surgery and 129 women at 15 months. Of these, only 9 women at 3 months and 4 women at 15 months had more than two missing items on the IES.

Total IES scores (PTSS) could be calculated for all but 25 women (*n*=3318) at 3 months, and all but 19 women at 15 months post surgery (*n*=2912). As shown in Table 1, the prevalence of severe PTSS was reduced from 20.1% at 3 months to 14.3% at 15 months (*P*<0.001). During the first year after surgery, 24.3% of the women had scores suggesting severe PTSS at one or both measurements. Of the women with severe PTSS at 3 months, 48.4% also had scores above the cutoff at 15 months. When analysing dropouts and women excluded at 15 months post surgery (*n*=424), the prevalence of severe PTSS at 3 months was higher in dropouts (23.1%) than in non-dropouts (19.6%). This difference was nonsignificant for severe PTSS (*χ*^*2*^=2.80; *P*=0.09), but significant for PTSS scores (*Z*=−2.32; *P*=0.02).

### Predictors

*Step 1: sociodemographic and comorbidity-related predictors* As seen in Table 2, the likelihood of severe PTSS increased with older age at 3 months, but not at 15 months, where the prevalence of older women (60–69 years) with severe PTSS had decreased sharply from 23.2 to 13.4%, whereas the percentage of young women (under 35 years) with severe PTSS doubled from 5.3 to 12.3%. Women with children had higher risk of severe PTSS at 3 months, but not at 15 months post surgery.

The socioeconomic status (SES) variables of education, occupational status, personal income, and household net wealth per person were all significant predictors of severe PTSS at 3 and 15 months post surgery in the age-adjusted analyses. Higher educational level was a substantial predictor of lower risk of severe PTSS at 3 and 15 months post surgery in both age-adjusted and fully adjusted analyses. For example, the age-adjusted odds of having severe PTSS at 3 months for women with a master's degree was only a third (OR=0.36) compared with women with <8 years of schooling. Lower income was consistently associated with higher risk at 3 and 15 months, whereas the effect of lower net wealth was most pronounced at 15 months. The SES variables of education, personal income, and household net wealth also showed to be significant predictors of severe PTSS at 15 months independent of 3 months PTSS and severe PTSS. Having a pre-cancer psychiatric history was consistently associated with substantially higher risk of cancer-related severe PTSS at both 3 and 15 months post surgery. The same pattern emerged for physical comorbidity. Psychiatric history, but not physical comorbidity, was also a significant predictor at 15 months in the 3 months-adjusted analyses.

*Step 2: clinical predictors* As seen in Table 3, lymph node involvement was consistently associated with markedly increased PTSS scores and risk of having severe PTSS, both at 3 and 15 months. A trend was also observed in the 3 months-adjusted analyses for nodal involvement to be predictive of sustained levels of severe PTSS at 15 months compared with no involvement (*P*=0.08, data not shown). After adjusting for age, mastectomy was significantly associated with higher risk of severe PTSS compared with lumpectomy at 15 months, but not at 3 months. After additional adjustment for nodal status however, type of surgery was no longer significant (*P*=0.89). The significant association between nodal status and PTSS was largely unaffected when adjusting for whether the women had undergone sentinel node procedure and the number of lymph nodes involved (data not shown). Having received or waiting to receive radiotherapy was associated with higher risk of severe PTSS at 3 months, but not at 15 months. But with additional adjustment for nodal status, this relationship was also nonsignificant (*P*=0.35), whereas nodal status retained its significance (*P*=0.01, data not shown). None of the clinical variables were significant in the 3 months-adjusted analyses (data not shown).

*Step 3: health behaviours, BMI, and PF* Poor PF at 3 months was the only independent predictor of higher PTSS scores and higher risk of severe PTSS both at 3 and 15 months post surgery in the fully adjusted analyses (Table 4). PF demonstrated an approximately linear relationship with severe PTSS. Nearly four times as many women with poor PF had severe PTSS, compared with those with the highest PF. The same pattern was observed in the 3 months-adjusted analysis of severe PTSS at 15 months (*P*=0.02, data not shown).

## Discussion

This study is to our knowledge the first nationwide prospective study of the prevalence of and potential risk factors for severe PTSS following breast cancer. The strengths of the study include: a large sample with an acceptable response rate at 3 months and a very high response rate at 15 months (94%), a prospective design using pre-cancer demographic factors and physical and psychiatric co-morbidity, and detailed data on disease-related, treatment-related, demographic, SES, and several important health-related variables. All data refers to specific time points or periods relative to the date of surgery.

When reviewing 16 studies of breast cancer patients published between 1995 and 2010, in which mean IES scores were reported, the mean intrusion scores reported varied from 1.24 to 22.0 and avoidance scores from 1.36 to 24.6. The highest scores were found in studies assessing cancer-related post-traumatic stress immediately after diagnosis or during treatment, whereas the lowest scores were found in samples of breast cancer survivors assessed up to 15 years post diagnosis. In addition to small samples (*N* from 44 to 283), there was considerable between- and within-study variability in the timing of assessment. However, when comparing our results with the scores found in the studies with assessment times most similar to ours (between 0 and 12 months), the intrusion and avoidance scores in our sample (10.1 and 10.0 at 3 months and 7.8 and 8.4 at 15 months) were similar or lower than the range of the scores found in these studies (intrusion: 9.1–14.0 and avoidance 12.2–15.0; a list of references can be obtained on request to the first author). At 3 months post surgery, we found a prevalence of severe PTSS of 20.1%. At 1 year later, the prevalence was reduced to 14.3%. One out of four women experienced severe PTSS during the first 15 months following surgery. In comparison, the lifetime prevalence of sufficiently severe PTSS to warrant a diagnosis of PTSD in general population studies has been found to be around 7% (Kessler *et al*, 2005). Our results thus confirm that a significant proportion of women experience severe breast cancer-related PTSS, and that a reduction in the prevalence of severe PTSS can be expected during the first year after surgery. However, approximately half of the women with severe PTSS at 3 months post surgery also had severe PTSS at 15 months, indicating that these women are at increased risk of persistent severe PTSS.

Our results, based on data from national registries with un-biased pre-cancer information, suggest that women with lower SES and previous physical or mental illness have a substantially higher risk of developing severe cancer-related PTSS than women with high social status and no previous illnesses. When adjusting for severe PTSS and PTSS at 3 months, women with high SES also improved more during the first year after surgery than women with lower SES, suggesting that PTSS may be more transient for women in high SES groups.

In the general population, the prevalence of sufficiently severe PTSS to warrant a diagnosis of PTSD is usually lower among older individuals compared with younger (Kessler *et al*, 2005). Likewise, younger cancer patients are generally more distressed after receiving their cancer diagnosis than older patients (Kangas *et al*, 2002). In contrast, the youngest women in our study had the lowest prevalence of severe PTSS at 3 months, whereas no differences were found for PTSS mean scores. At 15 months, no significant difference in severe PTSS was found between the youngest and the oldest patients, but younger patients now demonstrated significantly elevated PTSS mean scores. Although we have no clear explanation, the difference between our and previous results could stem from differences in timing and age characteristics of the studied samples.

Of the clinical factors, nodal status was a risk factor for severe PTSS at both 3 and 15 months post surgery. The prevalence of severe PTSS for women with >3 affected lymph nodes was markedly higher at 15 months (20.5%) than for women with no lymph node involvement (12.3%). Additional multivariate analyses confirmed that nodal involvement was the only independent clinical risk factor, suggesting that it is perceived disease severity rather than treatment that has a significant role in the development of severe cancer-related PTSS in early breast cancer. It may, therefore, be important to discuss the prognostic implications with the affected women to avoid potential misconceptions. It should also be taken into consideration that SES risk factors for severe PTSS such as lower education may also pose a communicative barrier.

Although some studies have indicated that alcohol consumption and smoking habits may be associated with PTSD (Rasmusson *et al*, 2006; Mcfarlane *et al*, 2009), these health behaviours were nonsignificant in the fully adjusted analyses. PF was clearly the strongest predictor of severe PTSS. Although evaluated after the cancer diagnosis, the age-stratified scores of PF in our study closely resemble national norms (Bjørner *et al*, 1997; Christensen *et al*, 2009). This suggests that the levels of PF found generally correspond to pre-cancer levels, and that the low PF therefore can be regarded as an independent risk factor of cancer-related severe PTSS, rather than a consequence hereof.

Although we have no clear explanation as to the association found between poor PF and greater cancer-related PTSS, possible biological, psychological, and social mechanisms could be considered in future studies. First, poor PF could be hypothesised to be at least partly related to various forms of disease activity, for example, inflammation. As proinflammatory cytokines have been shown to induce sickness behaviour and have been linked to psychological responses, for example, depression (Dantzer *et al*, 2008), it is possible that the link is mediated by proinflammatory cytokines. Perhaps more importantly, long-term cancer survivors have been shown to attribute problems, such as lack of energy and poor body image to their cancer (Phipps *et al*, 2008). A second, psychological explanation could therefore be that the women with poor PF, regardless of its aetiology, may have attributed their poor PF to their breast cancer, thus experiencing their disease as more serious, with subsequent greater cancer-related distress as result. Finally, poor PF may induce limitations in social function (Fialka-Moser *et al*, 2003), which in turn may be associated with poorer social support and subsequent increases in distress.

In line with previous findings (Van Hoof *et al*, 2009), our results show that the event itself may have a less clear role in relation to the development of severe PTSS than suggested in DSM-IV (American Psychiatric Association, 2000). It has been suggested that the nature of the event is more likely to be central as a predictor of post-traumatic stress in high-intensity stressors (e.g., direct experience of combat, torture, violent sexual assaults), whereas pre-existing individual risk factors may be the most important predictors of post-traumatic stress following relatively less extreme events (e.g., serious illness or bereavement; Van Hoof *et al*, 2009). By the above definition, primary breast cancer can be considered a less extreme traumatic stressor. Although one factor related to the intensity of the traumatic event, disease severity, was a risk factor for severe PTSS, our findings generally underline the importance of focusing on pre-existing individual risk factors, such as SES, previous mental or physical illness, and PF when screening for PTSS in breast cancer.

Although this study has several strengths, a few limitations should be mentioned. The IES score is based on symptoms experienced within the last 7 days. Other studies using other measures may have used longer time frames, and we may thus have underestimated the prevalence of PTSS in our sample. Furthermore, the participants in this study were generally younger, healthier, and had higher SES than non-participants resulting in a potential bias of underestimating the number of women suffering from severe PTSS. On the other hand, the IES only includes the DSM-IV criteria B and C (American Psychiatric Association, 2000), which may have lead us to overestimate the prevalence of sufficiently severe PTSS to suggest possible PTSD. Nevertheless, the IES cutoff of ⩾35 used in our study has previously shown good diagnostic performance (Neal *et al*, 1994; Wohlfarth *et al*, 2003).

## Conclusion

Although our measure of PTSS because of the large sample size was questionnaire-based rather than based on clinical interviews and a diagnosis of PTSD, therefore, could not be confirmed, our results show that breast cancer is a significant traumatic experience for a considerable proportion of disease-free women, even more than one year after surgery. Although nodal status was the most important clinical risk factor for severe breast cancer-related PTSS, pre-morbid conditions, such as SES, previous mental illness, and PF also were substantial risk factors. As most of these potential risk factors can be identified at the time of the diagnosis, this presents an opportunity to provide early preventive interventions for women at high risk of severe cancer-related PTSS.

## Figures and Tables

**Table 1:** Cancer-related PTSS and severe PTSS among women treated for primary breast cancer at 3 and 15 months post surgery

**IES (cancer related)**	**3 Months post surgery**	**15 Months post surgery**
*N*	3318	2912
Intrusive thoughts	Mean (s.d.): 10.1 (8.9)	Mean (s.d.): 7.8 (7.9)
	Median: 8	Median: 5
	Range: 0–35	Range: 0–35
	10–90 percentile 0–23	10–90 percentile 0–20
Avoidance	Mean (s.d.): 10.0 (8.8)	Mean (s.d.): 8.4 (8.7)
	Median: 8	Median: 6
	Range: 0–40	Range: 0–40
	10–90 percentile 0–23	10–90 percentile 0–21
IES total (PTSS)	Mean (s.d.): 20.1 (15.9)	Mean (s.d.): 16.2 (15.3)
	Median: 17	Median: 12
	Range: 0–75	Range: 0–71
	10–90 percentile 1–43	10–90 percentile 0–39
Severe PTSS: IES scores ⩾35 indicating severe symptomatology	666 (20.1 %)	415 (14.3%)

Abbreviations: IES=impact of event scale; PTSS=post-traumatic stress symptom.

**Table 2:** Pre-diagnostic socio-demographic and health status risk factors for cancer-related PTSS and severe PTSS^a^

	**3 Months post surgery**					**15 Months post surgery**						
		**PTSS**	**Severe PTSS**	**Severe PTSS age adjusted**			**PTSS**	**Severe PTSS**	**Severe PTSS age adjusted**		**Severe PTSS 3 months adjusted** b	
	***N***	**Mean (s.d.)**	**(%)**	**OR**	**(95% CI)**	***N***	**Mean (s.d.)**	**(%)**	**OR**	**(95% CI)**	**OR**	**(95 % CI)**
*Age*		*P*=0.71	*P*<0.001		*P*<0.001^c^		*P*<0.001	*P*=0.20		*P*=0.20^c^		*P*=0.07
26–35 years	95	17.05 (11.03)	6.3	1.00	(Referent)	73	17.36 (12.77)	12.3	1.00	(Referent)	1.00	(Referent)
36–49 years	900	19.76 (14.77)	17.2	**3.09**	1.33–7.18	773	17.39 (14.31)	12.9	1.06	0.51–2.19	0.57	0.26–1.26
50–59 years	1282	20.38 (16.17)	20.5	**3.83**	1.66–8.85	1126	16.68 (15.95)	16.0	1.35	0.66–2.77	0.69	0.32–1.49
60–69 years	1041	20.35 (16.69)	23.2	**4.49**	1.94–10.40	940	14.58 (15.47)	13.4	1.10	0.53–2.27	0.48	0.22–1.06
												
*Marital status*		*P*=0.47	*P*=0.15		*P*=0.44		*P*=0.04	*P*=0.33		*P*=0.32		*P*=0.17
Married or cohabiting	2537	19.90 (15.77)	19.4	1.00	(Referent)	2236	16.29 (15.35)	14.3	1.00	(Referent)	1.00	(Referent)
Divorced, separated or married – single	408	21.14 (15.97)	22.5	1.21	0.94–1.55	354	17.29 (15.70)	16.7	1.20	0.89–1.63	1.12	0.78–1.62
Widow – single	179	20.87 (16.78)	25.1	1.16	0.81–1.67	158	13.66 (14.90)	11.4	0.75	0.45–1.26	0.54	0.30–0.99
Unmarried – single	188	20.05 (15.79)	19.7	1.11	0.76–1.62	159	15.28 (14.67)	11.9	0.83	0.50–1.36	0.81	0.46–1.45
												
*Children*		*P*=0.16	*P*=0.007		*P*=0.01		*P*=0.25	*P*=0.08		*P*=0.08		*P*=0.53
No	384	18.70 (14.53)	14.8	1.00	(Referent)	326	15.02 (14.48)	11.0	1.00	(Referent)	1.00	(Referent)
Yes	2934	20.29 (16.01)	20.8	**1.45**	1.08–1.95	2586	16.36 (15.44)	14.7	1.38	0.96–1.98	1.15	0.75–1.75
												
*Education (ISCED 97 based)*		*P*=0.01	*P*<0.001		*P*<0.001		*P*=0.26	*P*<0.001		*P*<0.001		*P*=0.03
Lower secondary general (7 years)	539	22.42 (18.18)	27.8	1.00	(Referent)	466	16.55 (17.23)	18.0	1.00	(Referent)	1.00	(Referent)
Lower secondary general (8–10 years)	466	20.71 (16.65)	22.1	0.84	0.62–1.14	404	17.64 (16.29)	15.8	0.83	0.57–1.20	0.97	0.63–1.50
Upper secondary (11–13 years)	1317	20.41 (15.79)	20.3	**0.74**	0.58–0.95	1142	16.54 (15.47)	16.1	0.85	0.63–1.14	1.19	0.84–1.69
Tertiary < master degree (14–17 years)	797	18.29 (14.12)	15.2	**0.53**	0.40–0.71	718	14.97 (13.43)	9.2	**0.44**	0.31–0.64	0.68	0.45–1.03
Tertiary master degree (⩾18 years)	160	17.42 (12.24)	10.6	**0.36**	0.21–0.62	147	15.11 (13.43)	8.2	**0.39**	0.20–0.74	0.77	0.38–1.56
												
*Social status (ISCO-88 based)*		*P*<0.001	*P*<0.001		*P*<0.001		*P*=0.01	*P*<0.001		*P*<0.001		*P*=0.38
Top manager or employee – upper level	381	16.72 (12.55)	11.5	1.00	(Referent)	338	14.92 (12.78)	8.6	1.00	(Referent)	1.00	(Referent)
Employee – medium level	559	18.92 (14.60)	16.1	**1.48**	1.01–2.19	502	15.61 (13.88)	11.2	1.32	0.83–2.12	1.02	0.60–1.74
Employee – basic level	820	19.64 (15.32)	17.3	**1.61**	1.12–2.31	704	16.17 (15.09)	13.6	**1.67**	1.08–2.59	1.21	0.74–1.97
Employee – others or in education	333	20.63 (16.84)	22.2	**2.16**	1.44–3.25	288	17.78 (16.29)	18.8	**2.49**	1.54–4.03	1.56	0.89–2.72
Self-employed or assisting spouse	124	20.42 (15.88)	20.2	**1.88**	1.10–3.24	111	14.67 (14.30)	12.6	1.58	0.80–3.12	0.89	0.40–2.00
Unemployed, recipient of temporary allowance, cash or pre-retirement benefits, and so on	619	20.95 (16.50)	23.4	**2.18**	1.49–3.20	556	15.44 (15.84)	14.9	**2.03**	1.27–3.23	1.12	0.68–1.86
Old age pension	169	21.61 (17.83)	27.2	**2.52**	1.52–4.16	155	14.82 (15.44)	14.8	**2.16**	1.15–4.07	0.86	0.43–1.71
Recipients of early retirement pension, rehabilitation, or sickness benefits	306	24.60 (18.31)	32.4	**3.48**	2.32–5.20	252	20.55 (18.79)	23.4	**3.47**	2.12–5.66	1.53	0.87–2.68
												
*Personal income*		*P*=0.003	*P*<0.001		*P*<0.001		*P*=0.007	*P*<0.001		*P*<0.001		*P*=0.04
⩽20 000 $	499	22.35 (17.53)	28.1	1.00	(Referent)	436	18.67 (17.67)	20.0	1.00	(Referent)	1.00	(Referent)
>20 000 $ and ⩽30 000 $	618	21.22 (17.31)	23.9	0.83	0.63–1.08	544	15.06 (15.85)	14.7	**0.68**	0.49–0.96	0.68	0.45–1.04
>30 000 $ and ⩽40 000 $	688	20.94 (16.14)	21.9	0.78	0.59–1.03	600	17.11 (15.59)	17.7	0.82	0.59–1.13	1.04	0.71–1.54
>40 000 $ and ⩽55 000 $	879	18.68 (14.60)	15.4	**0.51**	0.39–0.68	775	15.29 (13.95)	10.3	**0.43**	0.31–0.61	**0.64**	0.43–0.95
>55 000 $	627	18.33 (13.88)	14.5	**0.48**	0.35–0.65	551	15.65 (14.05)	11.1	**0.47**	0.33–0.68	0.76	0.50–1.16
												
*Household net wealth (per person)*		*P*=0.15	*P*=0.39		*P*=0.03		*P*<0.001	*P*=0.001		*P*<0.001		*P*=0.02
<0 $	677	20.99 (16.41)	21.6	1.00	(Referent)	571	18.37 (15.97)	18.4	1.00	(Referent)	1.00	(Referent)
⩾0 $ and <20 000 $	598	20.38 (15.52)	20.9	0.87	0.66–1.15	533	17.20 (15.82)	14.3	**0.71**	0.51–0.98	0.73	0.50–1.08
⩾20 000 $ and <55 000 $	626	19.79 (15.95)	18.8	0.76	0.58–1.00	557	15.09 (14.94)	14.5	**0.72**	0.52–0.99	0.81	0.55–1.19
⩾55 000 $ and <120 000 $	736	20.54 (15.98)	21.1	0.82	0.63–1.07	645	16.19 (15.68)	14.6	**0.71**	0.52–0.97	0.77	0.53–1.11
⩾120 000 $	674	18.84 (15.32)	18.0	**0.64**	0.48–0.85	600	14.28 (13.85)	9.7	**0.43**	0.30–0.62	**0.50**	0.33–0.75
												
*Urbanicity (municipality size)*		*P*=0.86	*P*=0.95		*P*=0.92		*P*=0.94	*P*=0.45		*P*=0.47		*P*=0.59
<10 000	564	20.67 (16.22)	20.9	1.00	(Referent)	491	16.84 (15.92)	14.3	1.00	(Referent)	1.00	(Referent)
10 000–50 000	1201	20.10 (15.92)	20.1	0.92	0.72–1.18	1065	16.23 (15.62)	15.2	1.08	0.79–1.46	1.19	0.82–1.72
50 000–300 000	719	20.06 (15.93)	20.4	0.95	0.72–1.25	621	16.29 (15.49)	14.5	1.02	0.73–1.43	1.09	0.73–1.64
Copenhagen – suburbs	521	20.15 (15.58)	19.2	0.87	0.65–1.18	459	16.05 (14.66)	13.9	0.97	0.67–1.40	1.23	0.79–1.91
Copenhagen – center	307	19.20 (15.25)	19.2	0.91	0.64–1.29	271	15.14 (13.97)	10.7	0.72	0.46–1.14	0.84	0.49–1.44
												
*Ethnicity*		*P*=0.41	*P*=0.66		*P*=0.61		*P*=0.63	*P*=0.93		*P*=0.93		*P*=0.84
Not immigrant or descendant	3211	20.10 (15.91)	20.0	1.00	(Referent)	2821	16.20 (15.34)	14.3	1.00	(Referent)	1.00	(Referent)
Immigrant or descendant	101	20.55 (14.01)	21.8	1.13	0.70–1.84	86	16.83 (15.49)	14.0	0.97	0.52–1.81	1.07	0.54–2.15
												
*Psychiatric history*		*P*<0.001	*P*<0.001		*P*<0.001		*P*=0.002	*P*<0.001		*P*<0.001		*P*=0.008
No	3090	19.72 (15.67)	19.1	1.00	(Referent)	2728	15.92 (15.11)	13.5	1.00	(Referent)	1.00	(Referent)
Yes	228	25.38 (17.29)	32.9	**2.10**	1.57–2.81	184	20.45 (17.94)	26.1	**2.28**	1.61–3.22	**1.78**	1.16–2.72
												
*CCI*		*P*=0.002	*P*<0.001		*P*=0.001		*P*=0.03	*P*=0.001		*P*=0.002		*P*=0.26
No comorbidity	2955	19.78 (15.63)	19.2	1.00	(Referent)	2604	16.06 (15.27)	13.9	1.00	(Referent)	1.00	(Referent)
CCI score =1	296	22.27 (16.71)	26.0	**1.38**	1.05–1.83	251	16.66 (15.22)	14.7	1.07	0.74–1.55	0.84	0.55–1.31
CCI score >1	58	27.07 (19.81)	37.9	**2.26**	1.32–3.90	49	22.86 (18.24)	32.7	**3.01**	1.63–5.55	1.80	0.80–4.06

Abbreviations: CCI=Charlson comorbidity index; CI=confidence interval; IES=impact of events scale; ISCED=International Standard Classification of Education; ISCO-88= International Standard Classification of Occupation; OR=odds ratio; PTSS=post-traumatic stress symptom.

a PTSS=IES; severe PTSS=IES total score ⩾35.

b Adjusted for PTSS scores and severe PTSS measured at 3 months post surgery.

c Unadjusted.

Age- and baseline-adjusted ORs in bold differs significantly (95% CI) from the reference group (OR=1.00). Missing observations are not shown. As a consequence totals (*N*) differs slightly.

**Table 3:** Clinical risk factors for cancer-related PTSS and severe PTSS^a^

	**3 Months post surgery**							**15 Months post surgery**						
		**PTSS**	**Severe PTSS**	**Severe PTSS age adjusted**		**Severe PTSS fully adjusted** b			**PTSS**	**Severe PTSS**	**Severe PTSS age adjusted**		**Severe PTSS fully adjusted** c	
	***N***	**Mean (s.d.)**	**(%)**	**OR**	**(95% CI)**	**OR**	**(95% CI)**	***N***	**Mean (s.d.)**	**(%)**	**OR**	**(95% CI)**	**OR**	**(95 % CI)**
*Tumour size*		*P*=0.85	*P*=0.45		*P*=0.45		*P*=0.58		*P*=0.35	*P*=0.35		*P*=0.36		*P*=0.46
⩽*20 mm*	2009	19.99 (15.74)	19.5	1.00	(Referent)	1.00	(Referent)	1790	15.89 (15.19)	13.5	1.00	(Referent)	1.00	(Referent)
>20 mm and ⩽50 mm	1187	20.37 (16.14)	21.3	1.12	0.94–1.34	1.10	0.92–1.32	1019	16.83 (15.66)	15.5	1.17	0.94–1.46	1.15	0.92–1.44
>50 mm	101	20.33 (14.87)	19.8	1.05	0.63–1.73	1.04	0.62–1.73	85	16.51 (14.55)	14.1	1.05	0.56–1.97	0.98	0.52–1.86
														
*Nodal status*		*P*<0.001	*P*<0.001		*P*<0.001		*P*<0.001		*P*<0.001	*P*<0.001		*P*<0.001		*P*<0.001
0	1632	18.89 (15.40)	17.4	1.00	(Referent)	1.00	(Referent)	1467	15.28 (14.99)	12.3	1.00	(Referent)	1.00	(Referent)
1–3	1080	20.63 (15.83)	21.3	**1.30**	1.07–1.58	**1.30**	1.06–1.59	968	16.22 (15.20)	14.0	1.16	0.92–1.48	1.17	0.91–1.49
>3	594	22.58 (16.77)	25.4	**1.68**	1.34–2.11	**1.61**	1.28–2.03	468	19.24 (16.32)	20.5	**1.84**	1.40–2.42	**1.81**	1.37–2.40
														
*Tumour grade*		*P*=0.82	*P*=0.88		*P*=0.98		*P*=0.99		*P*=0.02	*P*=0.26		*P*=0.26		*P*=0.46
I	789	19.92 (16.21)	20.0	1.00	(Referent)	1.00	(Referent)	725	15.41 (15.33)	13.2	1.00	(Referent)	1.00	(Referent)
II	1189	20.35 (15.90)	20.4	1.04	0.83–1.30	1.01	0.80–1.27	1043	16.85 (15.86)	16.0	1.25	0.95–1.64	1.20	0.91–1.59
III	686	20.23 (15.60)	19.1	1.01	0.78–1.31	0.99	0.75–1.29	564	17.09 (14.82)	13.7	1.04	0.75–1.44	1.04	0.75–1.45
Non-ductal carcinoma	624	19.95 (15.66)	20.8	1.05	0.81–1.36	1.03	0.79–1.34	557	15.26 (14.87)	13.1	0.99	0.71–1.37	0.98	0.70–1.37
														
*ER/PR receptor status*		*P*=0.82	*P*=0.88		*P*=0.69		*P*=0.76		*P*=0.15	*P*=0.62		*P*=0.60		*P*=64
ER and PR negative	615	20.21 (15.78)	20.3	1.00	(Referent)	1.00	(Referent)	485	16.93 (15.10)	14.8	1.00	(Referent)	1.00	(Referent)
ER or PR positive	2672	20.09 (15.88)	20.1	0.96	0.77–1.19	0.97	0.77–1.21	2401	16.04 (15.37)	14.0	0.93	0.71–1.22	0.94	0.70–1.24
														
*Type of surgery*		*P*=0.55	*P*=0.47		*P*=0.49		*P*=0.87		*P*=0.31	*P*=0.04		*P*=0.04		*P*=0.12
Mastectomy	1795	20.36 (16.14)	20.6	1.00	(Referent)	1.00	(Referent)	1555	16.62 (15.79)	15.4	1.00	(Referent)	1.00	(Referent)
Lumpectomy	1515	19.82 (15.48)	19.5	0.94	0.79–1.12	0.99	0.83–1.18	1351	15.75 (14.78)	12.8	**0.81**	0.65–0.99	0.84	0.68–1.04
														
*Chemotherapy*		*P*=0.52	*P*=0.15		*P*=0.24		*P*=0.33		*P*<0.001	*P*=0.93		*P*=0.62		*P*=0.58
No chemotherapy	1871	20.18 (16.35)	21.0	1.00	(Referent)	1.00	(Referent)	1686	15.47 (15.83)	14.2	1.00	(Referent)	1.00	(Referent)
In treatment/treated	1435	20.05 (15.17)	19.0	1.13	0.92–1.39	1.11	0.90–1.38	1217	17.28 (14.56)	14.3	1.07	0.83–1.37	1.08	0.83–1.40
														
*Radiotherapy I*		*P*=0.004	*P*=0.02		*P*=0.004		*P*=0.005		*P*=0.09	*P*=0.43		*P*=0.42		*P*=0.31
No radiotherapy	680	18.51 (15.70)	16.5	1.00	(Referent)	1.00	(Referent)	619	15.50 (15.44)	13.2	1.00	(Referent)	1.00	(Referent)
To be treated after chemotherapy	1171	20.47 (15.09)	20.2	**1.56**	1.20–2.02	**1.56**	1.19–2.04							
Treated with radiotherapy	1456	20.59 (16.46)	21.8	**1.29**	1.02–1.65	**1.31**	1.02–1.67	2284	16.43 (15.30)	14.5	1.11	0.86–1.45	1.15	0.88–1.50
														
*Hormone therapy I*		*P*=0.22	*P*=0.02		*P*=0.54		*P*=0.69		*P*=0.06	*P*=0.17		*P*=0.16		*P*=0.26
No hormone therapy	1223	19.55 (15.77)	19.3	1.00	(Referent)	1.00	(Referent)	1029	15.62 (15.20)	13.0	1.00	(Referent)	1.00	(Referent)
To be treated after chemotherapy	821	19.95 (14.71)	17.9	1.15	0.88–1.49	1.12	0.86–1.47							
In treatment	1243	20.83 (16.67)	22.5	1.07	0.87–1.31	1.04	0.84–1.28	1861	16.58 (15.42)	14.9	1.17	0.94–1.46	1.14	0.91–1.43

Abbreviations: CI=confidence interval; ER=estrogen receptor; IES=impact of events scale; OR=odds ratio; PR=progesterone positive; PTSS=post-traumatic stress symptom.

a PTSS=IES; severe PTSS=IES total score ⩾35.

b Severe PTSS at 3 months post surgery fully adjusted for socio-demographic and health status variables related (*P*<0.25) to severe PTSS in the univariate and/or age-adjusted analyses (age, marital status, children, education, social status, personal income and household net wealth per person, psychiatric history, and comorbidity).

c Severe PTSS at 15 months post surgery fully adjusted for socio-demographic and health status variables related (*P*<0.25) to severe PTSS in the univariate and/or age-adjusted analyses (age, children, education, social status, personal income and household net wealth, psychiatric history, and comorbidity).

Age- and fully adjusted OR's in bold differs significantly (95% CI) from the reference group (OR=1.00). Missing observations are not shown. As a consequence totals (*N*) differs slightly.

**Table 4:** Self-reported health related risk factors for cancer-related PTSS and severe PTSS^a^

	**3 Months post surgery**							**15 Months post surgery**						
		**PTSS**	**Severe PTSS**	**Severe PTSS age adjusted**		**Severe PTSS fully adjusted** b			**PTSS**	**Severe PTSS**	**Severe PTSS age adjusted**		**Severe PTSS fully adjusted** c	
	***N***	**Mean (s.d.)**	**(%)**	**OR**	**(95% CI)**	**OR**	**(95% CI)**	***N***	**Mean (s.d.)**	**(%)**	**OR**	**(95% CI)**	**OR**	**(95 % CI)**
*Smoking status*		*P*=0.002	*P*<0.001		*P*<0.001		*P*=0.07		*P*=0.19	*P*=0.001		*P*=0.001		*P*=0.19
Never Smoker	1308	18.98 (15.48)	18.1	1.00	(Referent)	1.00	(Referent)	1155	15.44 (14.82)	12.9	1.00	(Referent)	1.00	(Referent)
Ex-smoker	989	19.71 (14.84)	17.9	1.00	0.81–1.24	1.05	0.84–1.31	879	15.83 (14.16)	12.5	0.97	0.74–1.26	1.03	0.79–1.36
1–9 per day	174	20.47 (16.27)	21.3	1.19	0.80–1.76	1.11	0.74–1.65	157	16.82 (15.14)	12.7	0.98	0.60–1.62	0.86	0.51–1.45
10–19 per day	458	20.39 (16.98)	26.4	**1.64**	1.27–2.11	1.44	1.11–1.88	388	17.37 (17.30)	17.3	**1.41**	1.03–1.93	1.19	0.85–1.66
⩾20 per day	333	22.30 (17.87)	24.6	**1.56**	1.17–2.08	1.30	0.95–1.77	283	18.72 (17.52)	21.2	**1.83**	1.31–2.56	1.50	1.04–2.15
														
*Alcohol*		*P*=0.02	*P*<0.001		*P*<0.001		*P*=0.01		*P*=0.04	*P*=0.001		*P*=0.001		*P*=0.09
Never drinker	331	20.42 (16.49)	21.8	1.00	(Referent)	1.00	(Referent)	282	16.96 (17.01)	21.3	1.00	(Referent)	1.00	(Referent)
Ex-drinker	176	24.23 (17.34)	30.7	**1.71**	1.13–2.59	**1.69**	1.09–2.62	148	18.96 (17.10)	20.3	0.94	0.58–1.55	0.89	0.53–1.51
<1 drink per day	1344	19.79 (15.70)	18.7	0.89	0.66–1.20	1.06	0.77–1.45	1187	16.15 (14.50)	12.5	**0.53**	0.38–0.74	0.62	0.44–0.89
⩾1 and <2 drinks per day	803	19.09 (14.91)	17.1	0.75	0.55–1.04	1.03	0.73–1.44	713	15.25 (15.06)	13.6	**0.58**	0.41–0.83	0.77	0.52–1.12
⩾2 and <3 drinks per day	385	20.01 (15.80)	21.3	0.96	0.67–1.38	1.33	0.91–1.94	333	15.42 (15.52)	12.3	**0.52**	0.34–0.80	0.67	0.42–1.06
⩾3 drinks per day	229	21.62 (16.97)	25.3	1.20	0.81–1.79	**1.68**	1.10–2.56	207	18.44 (16.69)	16.9	0.75	0.47–1.19	0.93	0.57–1.53
														
*Body mass index*		*P*=0.10	*P*=0.04		*P*=0.13		*P*=0.49		*P*=0.41	*P*=0.09		*P*=0.09		*P*=0.37
Normal weight (>18.5 and <25)	1898	19.59 (15.36)	18.3	1.00	(Referent)	1.00	(Referent)	1686	15.72 (14.89)	12.8	1.00	(Referent)	1.00	(Referent)
Underweight (⩽18.5)	84	18.04 (15.94)	19.0	1.02	0.58–1.78	0.87	0.49–1.55	71	15.23 (15.36)	11.3	0.87	0.41–1.84	0.74	0.34–1.61
Overweight (⩾25 and <30)	902	21.07 (16.23)	22.4	1.24	1.02–1.51	1.16	0.94–1.42	787	16.98 (15.76)	15.9	1.29	1.02–1.64	1.21	0.94–1.55
Obese or severely obese (⩾30)	375	20.72 (16.42)	22.1	1.23	0.94–1.61	1.01	0.76–1.35	321	16.82 (15.80)	16.5	1.35	0.98–1.88	1.06	0.75–1.51
														
*Physical function (SF-36 PF)*		*P*<0.001	*P*<0.001		*P*<0.001		*P*<0.001		*P*<0.001	*P*<0.001		*P*<0.001		*P*<0.001
100	537	14.25 (13.70)	9.7	1.00	(Referent)	1.00	(Referent)	476	11.34 (12.95)	6.7	1.00	(Referent)	1.00	(Referent)
>90 and <100	817	17.53 (13.91)	14.2	**1.57**	1.11–2.22	**1.63**	1.14–2.33	725	14.05 (13.25)	9.4	1.43	0.92–2.21	1.56	0.99–2.48
>80 and ⩽90	838	19.90 (15.04)	18.6	**2.16**	1.54–3.02	**2.18**	1.54–3.08	747	15.77 (14.97)	13.0	**2.07**	1.36–3.14	**2.17**	1.40–3.37
>70 and ⩽80	537	23.23 (16.11)	25.0	**3.12**	2.21–4.41	**3.12**	2.18–4.47	474	18.88 (15.72)	17.9	**3.03**	1.98–4.66	**3.24**	2.06–5.08
⩾0 and ⩽70	564	26.76 (18.08)	35.8	**5.00**	3.58–6.99	**4.31**	3.01–6.16	475	22.30 (17.91)	26.9	**5.19**	3.43–7.84	**4.94**	3.14–7.75

Abbreviations: CI=confidence interval; IES=impact of events scale; OR=odds ratio; PTSS=post-traumatic stress symptom.

a PTSS=IES; severe PTSS=IES total score ⩾35.

b Severe PTSS at 3 months post surgery fully adjusted for socio-demographic, health status, and clinical baseline variables related to severe PTSS (*P*<0.25) in the univariate and/or age-adjusted analyses (age, marital status, children, education, social status, personal income and household net-wealth per person, psychiatric history, comorbidity, nodal status, chemotherapy, radiotherapy, and hormone therapy).

c Severe PTSS at 15 months post surgery fully adjusted for socio-demographic, health status, and clinical baseline variables related to severe PTSS (*P*<0.25) in the univariate and/or age-adjusted analyses (age, children, education, social status, personal income and household net-wealth, psychiatric history, comorbidity), nodal status, surgery, and hormone therapy).

Age- and fully adjusted OR's in bold differs significantly (95% CI) from the reference group (OR=1.00). Missing observations are not shown. As a consequence totals (*N*) differs slightly. Smoking status, use of alcohol, weight, height, and physical function was measured by questionnaire at baseline.
